# Maslinic acid alleviates ischemia/reperfusion-induced inflammation by downregulation of NFκB-mediated adhesion molecule expression

**DOI:** 10.1038/s41598-019-42465-7

**Published:** 2019-04-16

**Authors:** Emmanuel Ampofo, Julian J. Berg, Michael D. Menger, Matthias W. Laschke

**Affiliations:** Institute for Clinical & Experimental Surgery, Saarland University, 66421 Homburg/Saar, Germany

## Abstract

Ischemia/reperfusion (I/R)-induced inflammation is associated with enhanced leukocyte rolling, adhesion and transmigration within the microcirculation. These steps are mediated by hypoxia-triggered signaling pathways, which upregulate adhesion molecule expression on endothelial cells and pericytes. We analyzed whether these cellular events are affected by maslinic acid (MA). Mitochondrial activity and viability of MA-exposed endothelial cells and pericytes were assessed by water-soluble tetrazolium (WST)-1 and lactate dehydrogenase (LDH) assays as well as Annexin V/propidium iodide (PI) stainings. Effects of MA on hypoxia and reoxygenation-induced expression of E-selectin, intercellular adhesion molecule (ICAM)-1 and vascular cell adhesion molecule (VCAM)-1 were determined by flow cytometry. The subcellular localization of the NFκB subunit p65 was analyzed by immunofluorescence and Western blot. I/R-induced leukocytic inflammation was studied in MA- and vehicle-treated mouse dorsal skinfold chambers by intravital fluorescence microscopy and immunohistochemistry. MA did not affect viability, but suppressed the mitochondrial activity of endothelial cells. Furthermore, MA reduced adhesion molecule expression on endothelial cells and pericytes due to an inhibitory action on NFκB signaling. Numbers of adherent and transmigrated leukocytes were lower in post-ischemic tissue of MA-treated mice when compared to vehicle-treated controls. In addition, MA affected reactive oxygen species (ROS) formation, resulting in a diminished oxidative DNA damage. Hence, MA represents an attractive compound for the establishment of novel therapeutic approaches against I/R-induced inflammation.

## Introduction

Ischemia/reperfusion (I/R) is associated with hypoxia-induced cytokine release, which promotes the binding of leukocytes to the microvascular endothelium and their transmigration across the endothelial lining into the surrounding tissue^1^. This process is mediated by specific adhesion molecules on endothelial cells^2^ and pericytes^3^. The expression of these surface proteins is strictly regulated by the NFκB signaling pathway^4^. Several studies have demonstrated that I/R initiates the activation of this pathway, resulting in the degradation of the IκB-complex and translocation of the main NFκB-subunit p65 into the nucleus^5–7^. This, in turn, induces the expression of the adhesion proteins E-selectin, intercellular adhesion molecule (ICAM)-1 and vascular cell adhesion molecule (VCAM)-1^8–10^.

The inhibition of the NFκB pathway is a promising approach to reduce inflammatory reactions. In the last years, several phytochemical compounds have been identified, which are capable of suppressing the activity of this pathway, such as apocyanin, curcumin and maslinic acid (MA)^11–13^. MA can be found in fruits and vegetables, particularly in the skin of olives^14^. Several studies reported potent anti-tumor^15,16^, anti-oxidant^17^ and anti-inflammatory^18–20^ effects of MA. These are mediated by inhibitory actions of MA on the activity of protein kinase (PK)C and the NFκB pathway and stimulatory actions on heme oxygenase (HO)-1 and endothelial nitric oxide synthase (eNOS), which suppresses the synthesis of important cytokines and reactive oxygen species (ROS)^13,21,22^.

These findings indicate that MA may also be capable of alleviating I/R-induced inflammation by downregulation of NFκB-triggered endothelial and pericyte adhesion molecule expression. To test this hypothesis, we first determined *in vitro* the viability, adhesion molecule expression and subcellular localization of p65 in vehicle- and MA-treated endothelial cells and pericytes under hypoxia and reoxygenation. In addition, we analyzed *in vivo* leukocyte rolling, adhesion and transmigration in dorsal skinfold chambers of vehicle- and MA-treated mice, which were exposed to I/R.

## Results

### Effect of MA on mitochondrial activity and viability of endothelial cells

**Figure 1:**
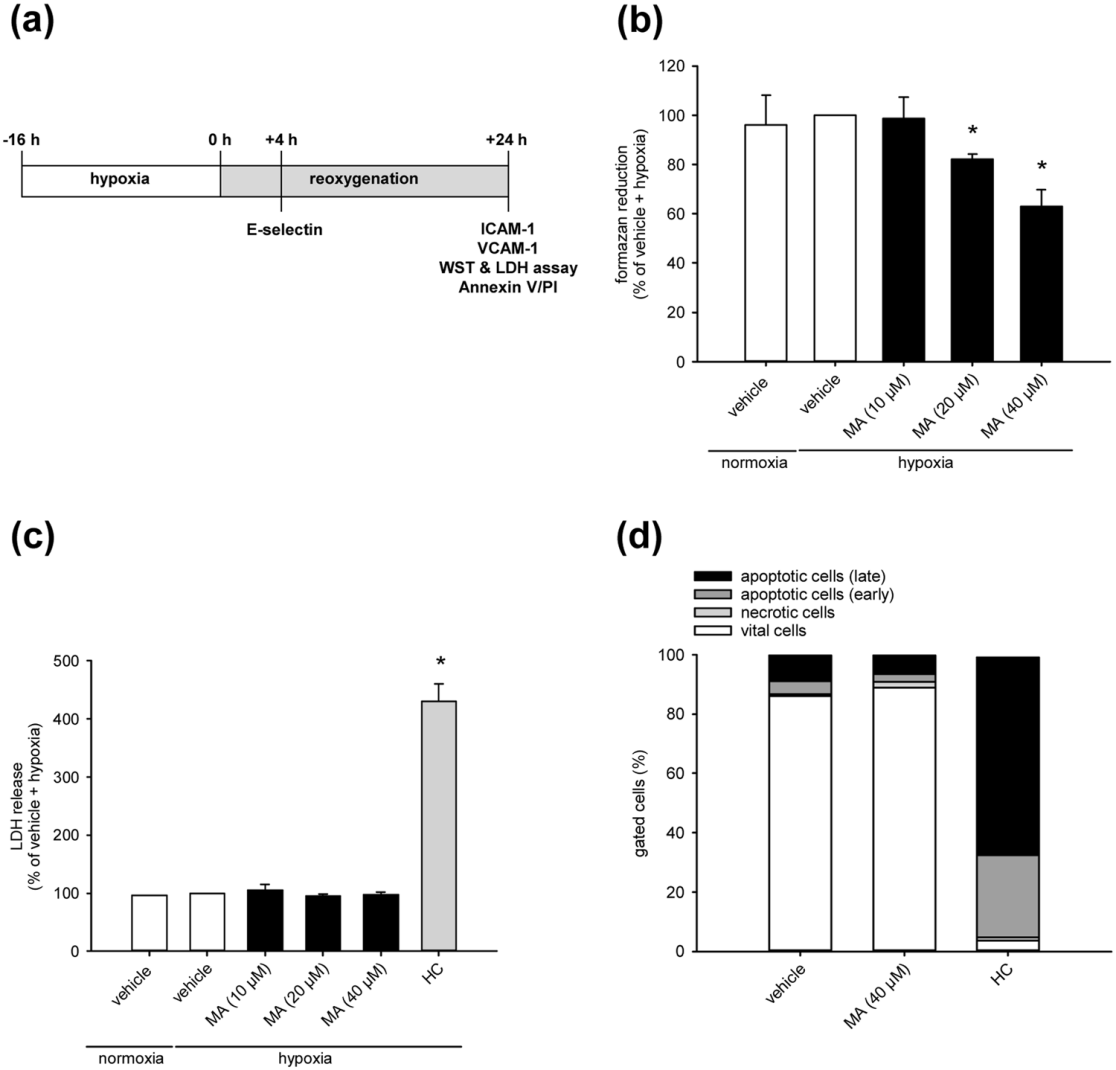
Effect of MA on mitochondrial activity and viability of endothelial cells. (**a**) Schematic illustration of the experimental hypoxia and reoxygenation setup to simulate I/R *in vitro*. (**b**,**c**) HDMEC were treated with vehicle (DMSO; white bars) or the indicated concentrations of MA (black bars) and cultivated for 16 h under normoxia or hypoxia. The cells were reoxygenated for 24 h and their mitochondrial activity and viability was assessed by a WST-1 (**b**) and a LDH assay (**c**). Hypoxia-exposed vehicle-treated cells were used as control and set 100% (n = 5). Lysed cells were used as positive control for the LDH assay (c, grey bar) (n = 5). Mean ± SD. *P < 0.05 vs. vehicle + hypoxia. (**d**) HDMEC were exposed to vehicle (DMSO) or MA (40 µM) and cultivated for 16 h under hypoxia. The cells were then reoxygenated for 24 h and Annexin V/PI-positive cells (% of gated cells) were assessed by flow cytometry (n = 5).

Vehicle- and MA-treated human dermal microvascular endothelial cells (HDMEC) were cultivated under hypoxia and reoxygenation to mimic I/R *in vitro* (Fig. 1a). Thereafter, we performed a water-soluble tetrazolium (WST)-1 assay and found that concentrations of MA ranging from 20–40 µM reduce the mitochondrial activity of HDMEC when compared to vehicle-treated controls (Fig. 1b). In contrast, MA treatment did neither result in an increase of lactate dehydrogenase (LDH) release (Fig. 1c) nor in a higher number of Annexin V/propidium iodide (PI)-positive cells (Fig. 1d). This indicates that MA dose-dependently affects the mitochondrial activity of endothelial cells without inducing apoptotic or necrotic cell death.

### Effect of MA on the expression of endothelial surface adhesion proteins and NFκB signaling

**Figure 2:**
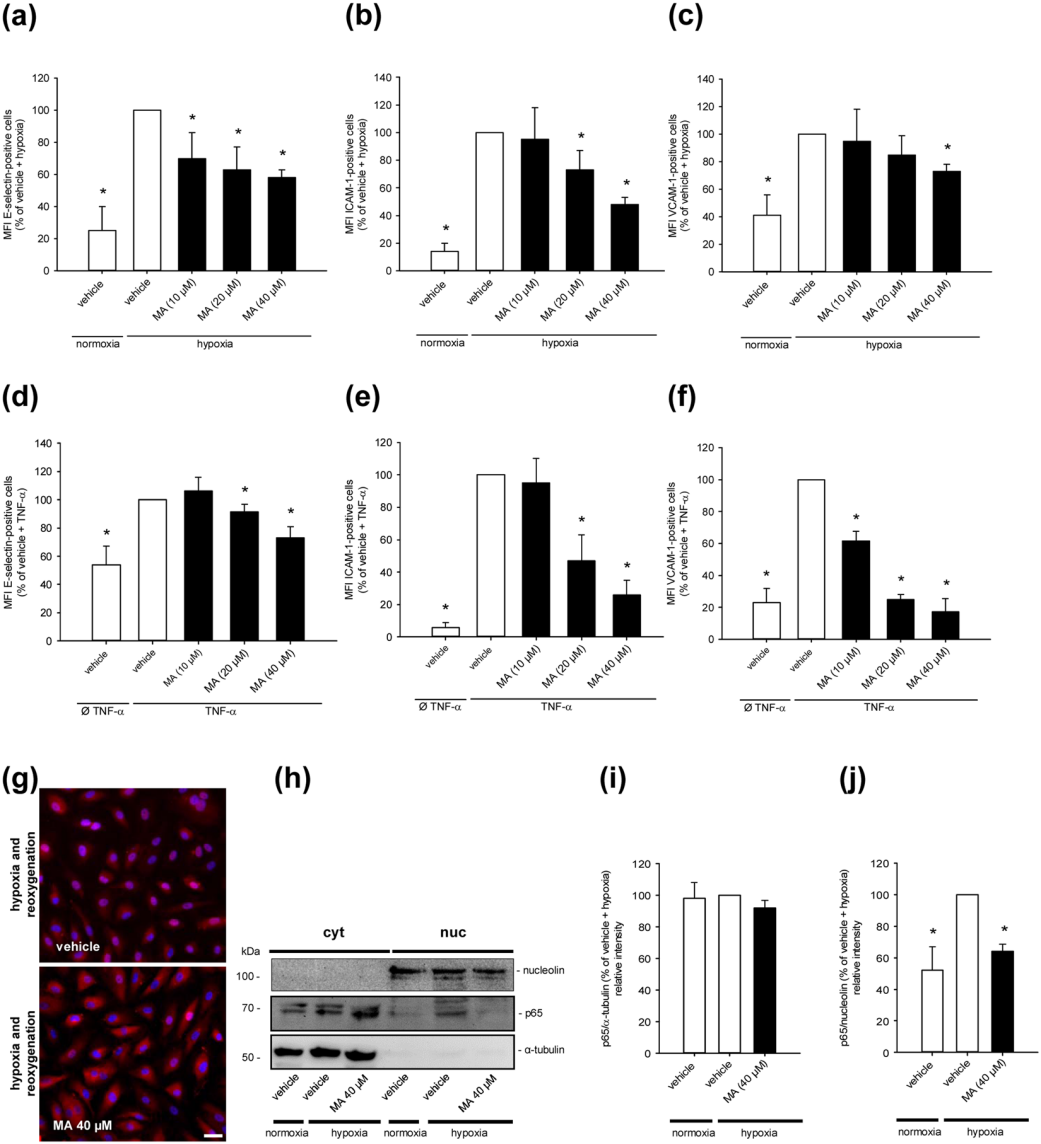
Effect of MA on the expression of endothelial surface adhesion proteins and NFκB signaling. (**a–c**) HDMEC were exposed to vehicle (DMSO; white bars) or the indicated concentrations of MA (black bars) and cultivated for 16 h under normoxia or hypoxia. The cells were then reoxygenated for 4 h or 24 h. The MFI of E-selectin (**a**) was assessed by flow cytometry after 4 h and the MFI of ICAM-1 (**b**) and VCAM-1 (**c**) was assessed by flow cytometry after 24 h. Hypoxia-exposed vehicle-treated cells were used as control and set 100% (n = 5). Mean ± SD. *P < 0.05 vs. vehicle + hypoxia. (**d–f** ) HDMEC were exposed to vehicle (DMSO; white bars) or the indicated concentrations of MA (black bars) and cultivated for 24 h with or without TNF-α. After 24 h, the expression of E-selectin (**d**), ICAM-1 (**e**) and VCAM-1 (**f**) was assessed by flow cytometry. Vehicle-treated, TNF-α-exposed cells were used as control and set 100% (n = 5). Mean ± SD. *P < 0.05 vs. vehicle + TNF-α. (**g**) Immunofluorescence images of vehicle (DMSO)- or MA (40 µM)-treated HDMEC, which were cultivated for 16 h under hypoxia. Thereafter, the cells were reoxygenated for 4 h and p65 was detected with an anti-p65 antibody. Scale bar: 50 µm. Images are representative of experiments conducted in triplicate. (**h**) HDMEC were exposed to vehicle (DMSO) or MA (40 µM) and cultivated as described in (**g**). The cytoplasmic and nuclear localization of p65 was analyzed by Western blot. Bands were cropped from different parts of the same gel. (**i**,**j**) p65/α-tubulin (% of vehicle + hypoxia) (**i**) and p65/nucleolin (% of vehicle + hypoxia) (**j**), as assessed by quantitative analysis of Western blots (n = 5). Mean ± SD. *P < 0.05 vs. vehicle + hypoxia.

Next, we analyzed the expression of E-selectin, ICAM-1 and VCAM-1 during hypoxia and reoxygenation. Hypoxia and reoxygenation significantly increased the expression of these adhesion surface molecules (Fig. 2a–c). Of interest, MA reduced dose-dependently the expression of E-selectin, ICAM-1 and VCAM-1 when compared to vehicle-treated controls (Fig. 2a–c). To investigate whether this effect is mediated by the inhibitory action of MA on NFκB, the cells were stimulated with tumor necrosis factor (TNF)-α, which specifically activates this pathway. This control experiment demonstrated indeed that the stimulatory effect of TNF-α on endothelial adhesion molecule expression is also suppressed by MA (Fig. 2d-f). Accordingly, we further determined the subcellular localization of the NFκB subunit p65 in HDMEC. Immunofluorescence analyses demonstrated an increased nuclear staining of p65 in the cells after hypoxia and reoxygenation (Fig. 2g). MA treatment attenuated the nuclear localization of the transcription factor when compared to vehicle-treated controls. These results could be confirmed by additional Western blot analyses, demonstrating an increased nuclear protein level of p65 in vehicle-treated HDMEC after hypoxia and reoxygenation and a significant reduction by MA exposure (Fig. 2h–j).

### Effect of MA on mitochondrial activity, viability, ICAM-1 expression and NFκB signaling in pericytes

**Figure 3:**
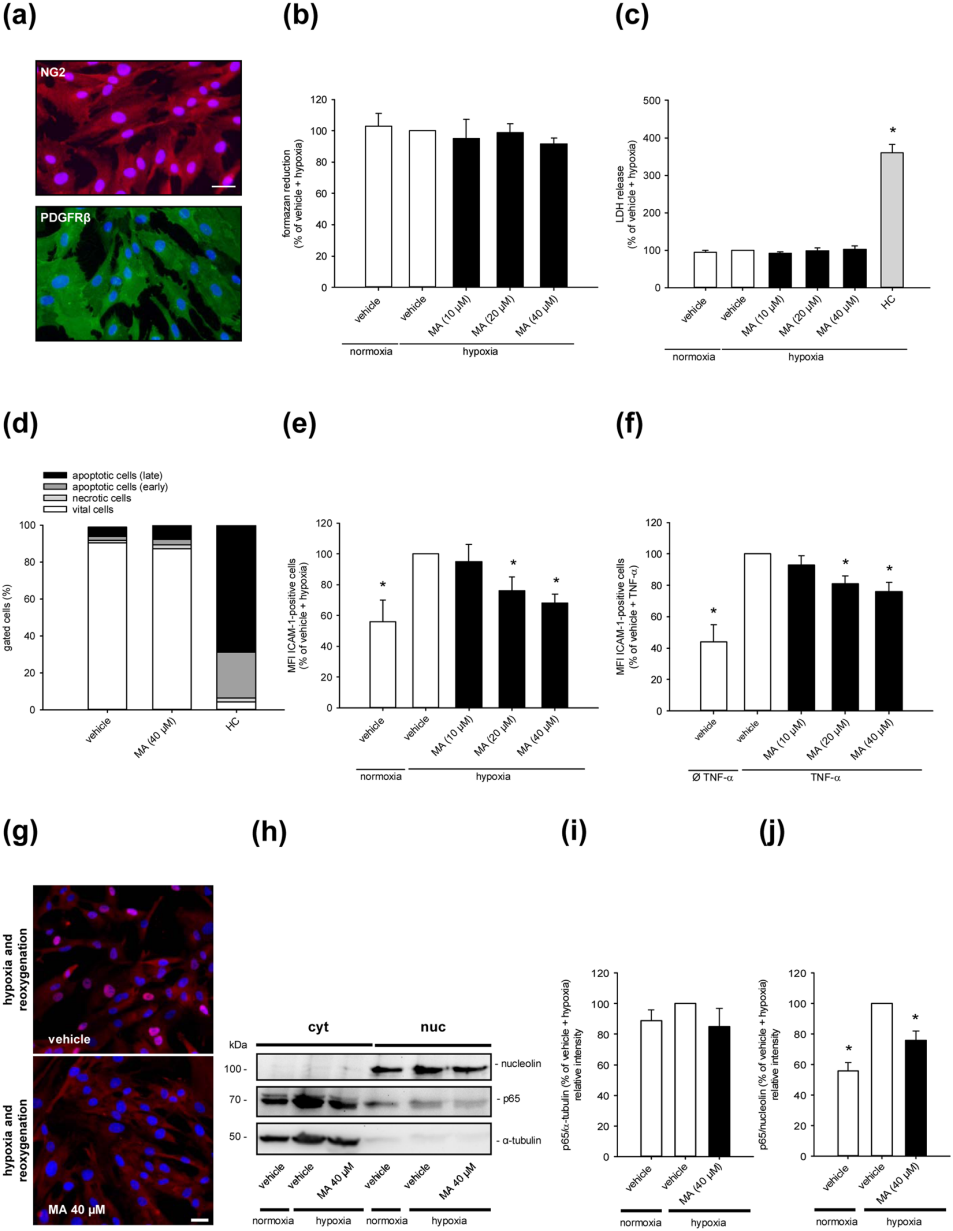
Effect of MA on mitochondrial activity, viability, ICAM-1 expression and NFκB signaling in pericytes. (**a**) Pericytes were characterized by NG2 and PDGFR-β expression. Scale bar: 50 µm. (**b**,**c**) Pericytes were treated with vehicle (DMSO; white bars) or the indicated concentrations of MA (black bars) and cultivated for 16 h under normoxia or hypoxia. The cells were reoxygenated for 24 h and their mitochondrial activity and viability were assessed by a WST-1 (**b**) and a LDH assay (**c**). The hypoxia-exposed vehicle-treated cells were used as control and set 100% (n = 5). Lysed cells were used as positive control for the LDH assay (c, grey bar) (n = 5). Mean ± SD. *P < 0.05 vs. vehicle + hypoxia. (**d**) Pericytes were exposed to vehicle (DMSO) or MA (40 µM) and cultivated for 16 h under hypoxia. The cells were then reoxygenated for 24 h and Annexin V/PI-positive cells (% of gated cells) were assessed by flow cytometry (n = 5). **(e)** Pericytes were cultivated as described in (**b**,**c**) and the MFI of ICAM-1 was assessed by flow cytometry. Hypoxia-exposed vehicle-treated cells were used as control and set 100% (n = 5). Mean ± SD. *P < 0.05 vs. vehicle + hypoxia. (**f**) Pericytes were exposed to vehicle (DMSO; white bars) or the indicated concentrations of MA (black bars) and cultivated for 24 h with or without TNF-α. The expression of ICAM-1 was assessed by flow cytometry. Vehicle-treated, TNF-α-exposed cells were used as control and set 100% (n = 5). Mean ± SD. *P < 0.05 vs. vehicle + TNF-α. (**g**) Immunofluorescence images of vehicle (DMSO)- or MA (40 µM)-treated pericytes, which were cultivated for 16 h under hypoxia and reoxygenated for 4 h. p65 was detected with an anti-p65 antibody. Scale bar: 50 µm. Images are representative of experiments conducted in triplicate. (**h**) Pericytes were exposed to vehicle (DMSO) or MA (40 µM) and cultivated as described in (**g**). The cytoplasmic and nuclear localization of p65 was analyzed by Western blot. Bands were cropped from different parts of the same gel. (**i**,**j**) p65/α-tubulin (% of vehicle + hypoxia) (**i**) and p65/nucleolin (% of vehicle + hypoxia) (**j**), as assessed by quantitative analysis of Western blots (n = 5). Mean ± SD. *P < 0.05 vs. vehicle + hypoxia.

Besides endothelial cells, pericytes also crucially contribute to neutrophil crawling via the adhesion molecule ICAM-1^3^. Therefore, we additionally assessed the effects of MA treatment on the viability and protein expression of human pericytes. For this purpose, they were first characterized by immunofluorescent detection of the typical surface proteins platelet-derived growth factor receptor (PDGFR)-β and nerve/glial antigen (NG)2 (Fig. 3a). Next, we could demonstrate that neither hypoxia and reoxygenation nor MA affect the mitochondrial activity of the cells and do not induce apoptotic or necrotic cell death (Fig. 3b–d). In addition, according to the results from the endothelial cell assays, hypoxia and reoxygenation- and TNF-α-induced ICAM-1 expression was also dose-dependently reduced by MA in pericytes when compared to vehicle-treated controls (Fig. 3e,f). Further experiments with hypoxia and reoxygenation revealed that this was due to a prevention of shuttling of p65 into the nucleus (Fig. 3g–j).

### Effect of MA on I/R-induced leukocyte-endothelial cell interaction

**Figure 4:**
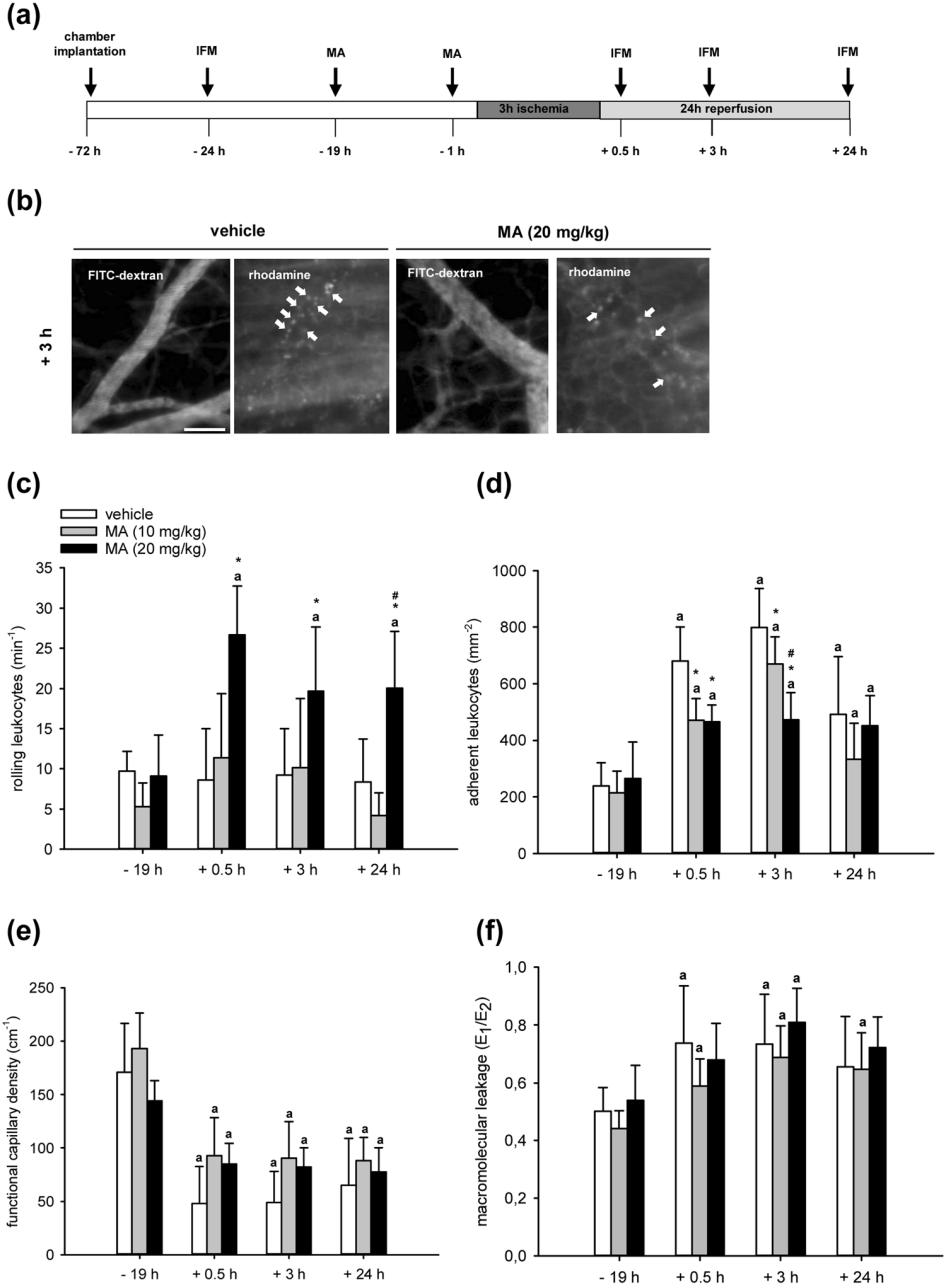
Effect of MA on I/R-induced leukocyte-endothelial cell interaction. (**a**) Schematic illustration of the experimental I/R setup *in vivo*. (**b**) Intravital fluorescence microscopic images of collecting venules in dorsal skinfold chambers of vehicle-treated and MA (20 mg/kg)-treated mice after 3 h ischemia and 3 h reperfusion. The plasma marker 5% FITC-labeled dextran was used for the visualization of microvessels. Leukocytes (white arrows) were stained *in vivo* by rhodamine 6 G. Scale bar: 50 μm. (**c–f**) Mice with dorsal skinfold chambers were treated with vehicle (DMSO; white bars), low dose MA (10 mg/kg; grey bars) and high dose MA (20 mg/kg; black bars). Rolling leukocytes (min^−1^) (c), adherent leukocytes (mm^−2^) (**d**), functional capillary density (cm^−1^) (**e**) and macromolecular leakage (E1/E2) (**f**) were assessed by intravital fluorescence microscopy and computer-assisted image analysis 19 h before and 0.5 h, 3 h and 24 h after 3 h ischemia (n = 8 per group). Mean ± SD. ^a^P < 0.05 vs. −19 h (baseline) in each individual group; *P < 0.05 vs. vehicle. ^#^P < 0.05 vs. low dose MA.

Based on our *in vitro* findings, we finally investigated the effects of MA on I/R-induced inflammation within a mouse dorsal skinfold chamber model (Fig. 4a,b). For this purpose, the animals were treated with doses of 10 mg/kg MA or 20 mg/kg MA i.p., which have already been shown to be effective *in vivo*^23^. Moreover, we treated the animals 19 h and 1 h before the induction of ischemia to ensure the inhibition of as many potential MA targets as possible.

First, we determined microhemodynamic parameters of those venules, which were used for the *in vivo* analysis of leukocyte-endothelial cell interactions by means of intravital fluorescence microscopy. I/R induced vasodilation in reperfused postcapillary and collecting venules in dorsal skinfold chambers of vehicle- and MA-treated mice, whereas centerline red blood cell (RBC) velocity, volumetric blood flow and wall shear rate of these vessels were not affected by I/R (Table 1). Importantly, MA did not influence these parameters when compared to vehicle-treated mice (Table 1), indicating that the interaction of leukocytes with the microvascular endothelium could be investigated under comparable microhemodynamic conditions.

**Table 1:** Diameter (µm), centerline RBC velocity (µm/s), volumetric blood flow (pL/s) and wall shear rate (s^−1^) of postcapillary and collecting venules in dorsal skinfold chambers of vehicle-treated, low dose MA (10 mg/kg)-treated and high dose MA (20 mg/kg)-treated mice 19 h before as well as 0.5 h, 3 h and 24 h after ischemia (n = 8 per group).

	−19 h	+0.5 h	+3 h	+ 24 h
**diameter (µm)**				
vehicle	26.0 ± 3.4	32.3 ± 2.2^a^	30.9 ± 2.4^a^	31.8 ± 4.0^a^
MA (10 mg/kg)	22.9 ± 2.9	29.7 ± 3.6^a^	28.1 ± 2.3^a^	26.7 ± 1.5^a^
MA (20 mg/kg)	25.5 ± 2.8	32.3 ± 2.2^a^	31.8 ± 2.9^a^	30.3 ± 2.8^a^
**centerline RBC velocity (µm/s)**				
vehicle	308.8 ± 142.4	186.2 ± 117.8	227.9 ± 127.6	243.6 ± 129.1
MA (10 mg/kg)	270.4 ± 101.4	265.4 ± 175.5	239.5 ± 134.2	186.6 ± 70.2
MA (20 mg/kg)	533.8 ± 166.9	463.9 ± 184.5	388.1 ± 147.1	348.8 ± 117.7
**volumetric blood flow (pL/s)**				
vehicle	103.0 ± 57.5	95.4 ± 58.1	109.7 ± 57.9	110.7 ± 60.0
MA (10 mg/kg)	70.4 ± 32.4	118.2 ± 59.5	93.6 ± 37.2	57.8 ± 35.0
MA (20 mg/kg)	170.7 ± 66.2	234.8 ± 85.4	186.6 ± 34.3	158.5 ± 47.4
**wall shear rate (s** ^**−1**^ **)**				
vehicle	93.1 ± 53.0	45.8 ± 28.4	61.2 ± 33.1	65.9 ± 43.2
MA (10 mg/kg)	94.9 ± 39.4	71.6 ± 41.2	68.4 ± 21.9	55.8 ± 23.8
MA (20 mg/kg)	170.1 ± 53.9	116.2 ± 40.2	100.5 ± 34.4	88.2 ± 20.9

Mean ± SD. ^a^P < 0.05 vs. −19 h (baseline) in each individual group.

I/R did not affect the rolling of leukocytes in vehicle-treated mice and in animals receiving low dose MA (10 mg/kg) when compared to baseline values (Fig. 4c). In contrast, treatment of mice with high dose MA (20 mg/kg) significantly increased the number of rolling leukocytes during post-ischemic reperfusion (Fig. 4c). In all groups, the number of adherent leukocytes was significantly elevated after I/R when compared to baseline conditions (Fig. 4d). Interestingly, MA treatment significantly reduced the I/R-induced increase of leukocyte adherence in venules during the early post-ischemic reperfusion period (Fig. 4b,d).

We further analyzed the effects of I/R on the functional capillary density and macromolecular leakage within the dorsal skinfold chambers. As expected, I/R significantly decreased the functional capillary density and increased the macromolecular leakage (Fig. 4e,f). However, these parameters were not affected by MA (Fig. 4e,f).

**Figure 5:**
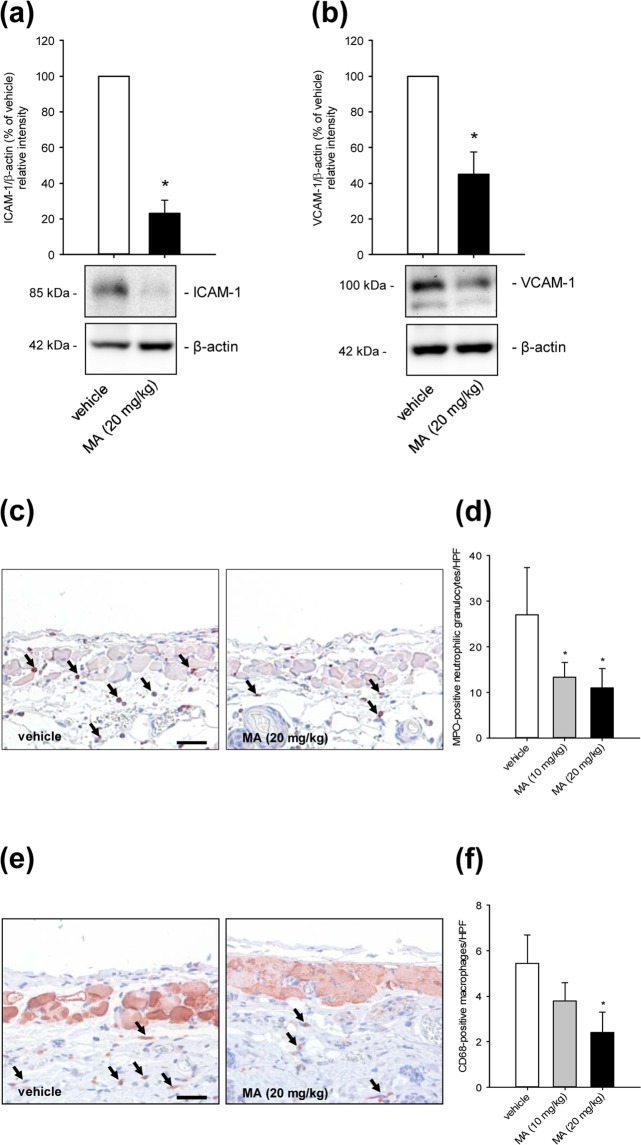
Effect of MA on ICAM-1 and VCAM-1 expression and immune cell recruitment in I/R-exposed tissue. (**a**,**b**) The expression of ICAM-1 (**a**) and VCAM-1 (**b**) in extracts of dorsal skinfold chamber tissue from vehicle-treated (DMSO; white bars) and MA (20 mg/kg)-treated animals (3 h after reperfusion; black bars) was analyzed by Western blot. Bands were cropped from different parts of the same gel. ICAM-1/β-actin (% of vehicle) (**a**) and VCAM-1/β-actin (% of vehicle) (**b**), as assessed by quantitative analysis of Western blots (n = 3). Mean ± SD. *P < 0.05 vs. vehicle. (**c**,**e**) Immunohistochemical detection of transmigrated MPO-positive neutrophilic granulocytes (black arrows, c) and CD68-positive macrophages (black arrows, e) in dorsal skinfold chambers of vehicle-treated and MA (20 mg/kg)-treated animals after 3 h ischemia and 24 h reperfusion. Scale bar: 50 µm. (**d**,**f**) Transmigrated MPO-positive neutrophilic granulocytes (**d**) and CD68-positive macrophages (**f**) (per HPF) in dorsal skinfold chambers of vehicle-treated (DMSO; white bars), low dose MA (10 mg/kg)-treated (grey bars) and high dose MA (20 mg/kg)-treated mice (black bars) were assessed by quantitative analysis of immunohistochemical sections (n = 8 per group). Mean ± SD. *P < 0.05 vs. vehicle.

Next, we investigated the expression of ICAM-1 and VCAM-1 in the dorsal skinfold chamber tissue after 3 h ischemia and 3 h reperfusion by means of Western blot (Fig. 5a,b). In line with our *in vitro* results, we found that the expression of these adhesion molecules was significantly reduced in high dose MA-treated animals. In addition, we assessed the number of immune cells infiltrating the tissue of the dorsal skinfold chamber by means of immunohistochemistry. Our analyses revealed that the number of myeloperoxidase (MPO)-positive neutrophilic granulocytes and CD68-positive macrophages was significantly lower in MA-treated mice after 3 h ischemia and 24 h reperfusion when compared to controls (Fig. 5c–f).

**Figure 6:**
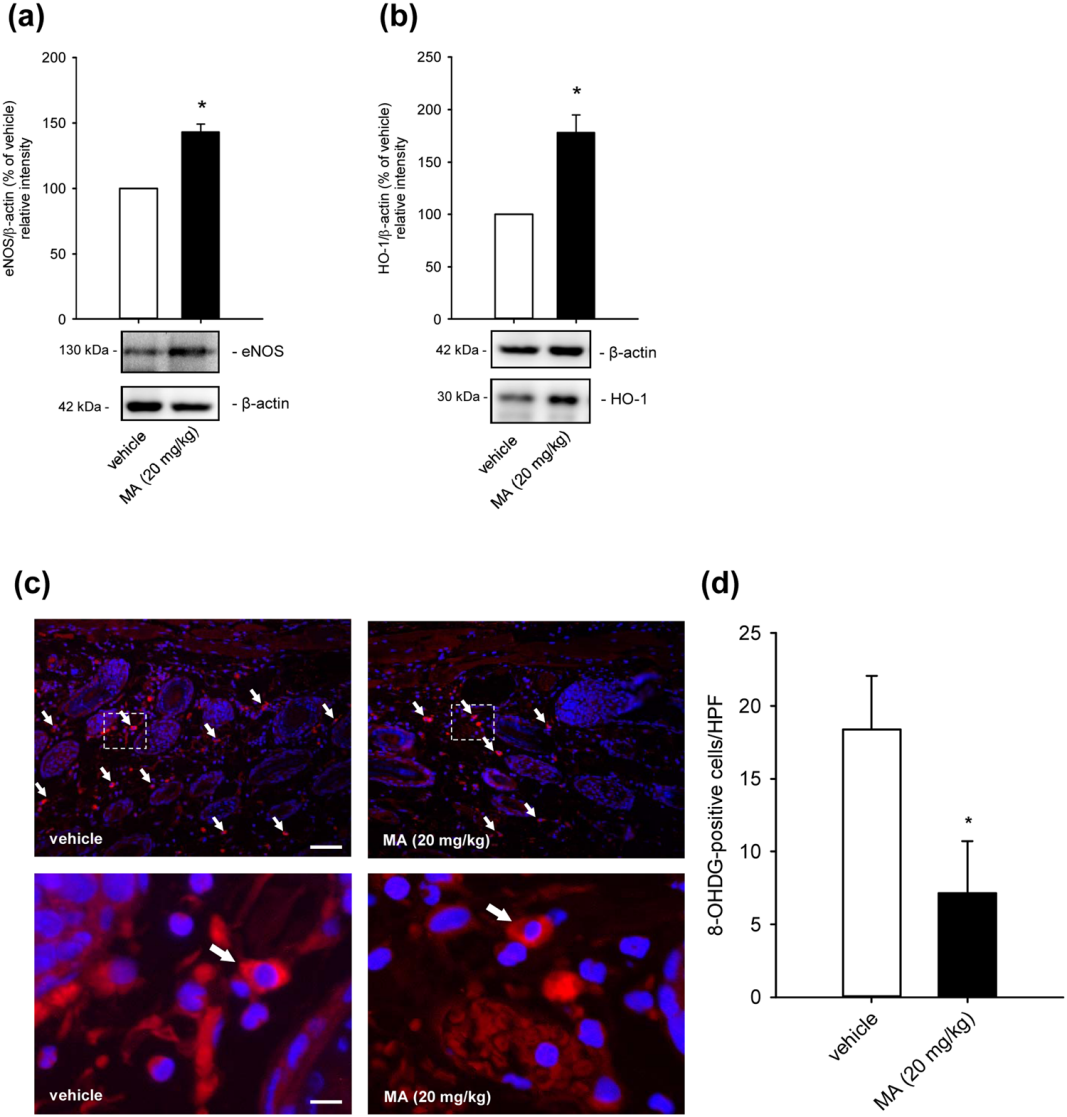
Effect of MA on anti-oxidative enzyme expression and oxidative DNA damage. (**a**,**b**) The expression of eNOS (**a**) and HO-1 (**b**) in extracts of dorsal skinfold chamber tissue from vehicle-treated (DMSO; white bars) and MA (20 mg/kg)-treated animals (black bars) after 3 h ischemia and 3 h reperfusion was analyzed by Western blot. Bands were cropped from different parts of the same gel. eNOS/β-actin (% of vehicle) (**a**) and HO-1/β-actin (% of vehicle) (**b**), as assessed by quantitative analysis of Western blots (n = 3). Mean ± SD. *P < 0.05 vs. vehicle. (**c**) Immunohistochemical detection of 8-OHDG-positive cells (white arrows) in dorsal skinfold chambers of vehicle-treated and MA (20 mg/kg)-treated animals after 3 h ischemia and 24 h reperfusion. Scale bar: 75 µm. Dashed boxes (lower panel) show 8-OHDG-stained cells in a higher magnification. Scale bar: 10 µm. (**d**) 8-OHDG-positive cells (per HPF) in dorsal skinfold chambers of vehicle-treated (DMSO; white bar), and high dose MA (20 mg/kg)-treated mice (black bar) were assessed by quantitative analysis of immunohistochemical sections (n = 8 per group). Mean ± SD. *P < 0.05 vs. vehicle.

We further analyzed the expression of the NO-producing enzyme eNOS and the heat shock protein HO-1 within the tissue of vehicle- and MA-treated dorsal skinfold chambers. After 3 h ischemia and 3 h reperfusion, MA significantly induced the expression of the anti-oxidative proteins (Fig. 6a,b). Moreover, we investigated the effect of MA on the oxidative DNA degradation by means of 8-hydroxydeoxyguanosine (8-OHDG) staining after 3 h ischemia and 24 h reperfusion. Of note, the number of 8-OHDG-positive cells was markedly diminished in MA-treated animals when compared to controls (Fig. 6c,d).

## Discussion

The phytochemical MA is a pentacyclic triterpene with a broad spectrum of biological activities^24^. The anti-inflammatory action of this compound has been extensively investigated^16,25^. However, the effects of MA on leukocyte-endothelial cell interactions have not been analyzed so far. In our study, we show that MA suppresses I/R-induced leukocyte adhesion and transmigration into the post-ischemic tissue.

In the last decade, most studies focused on the anti-cancer activity of MA, because this compound exerts anti-proliferative and pro-apoptotic effects^26–28^. Of interest, doses of more than 100 µM MA have been shown to suppress cell growth and to induce apoptosis, whereas low doses of <100 µM solely affect the proliferation rate of tumor cells^29^. In this study, we assessed cell proliferation by measuring mitochondrial activity using a WST-1 assay. This assay is based on the cleavage of tetrazolium salt by mitochondrial dehydrogenases to form formazan in viable cells. This correlates with the number of vital cells and, thus, is frequently used for the indirect measurement of cell proliferation. We found that treatment with 20–40 µM MA decreases the mitochondrial activity of endothelial cells. In contrast, these doses of MA did not affect the proliferation of pericytes. This contradictory result may be explained by the fact that pericytes exhibit a reduced proliferation rate when compared to HDMEC. Thus, HDMEC may be more affected by the anti-proliferative effect of MA than pericytes. In line with this view, Reyes *et al*.^30^ reported that MA particularly induces G0/G1 arrest in high proliferative cancer cells.

Our *in vitro* analyses revealed that MA dose-dependently diminishes the level of E-selectin, ICAM-1 and VCAM-1 on endothelial cells. These surface adhesion proteins are important for the interaction with leukocytes. E-selectin mediates leukocyte rolling along the microvascular endothelium^31^, whereas ICAM-1 and VCAM-1 are essential for firm leukocyte adhesion and transmigration^2,32,33^. Beside endothelial cells, pericytes are also crucially involved in the process of leukocyte extravasation^3,34,35^. In fact, ICAM-1 on pericytes binds to its leukocytic counter receptors lymphocyte function-associated antigen (LFA)-1 and macrophage antigen (Mac)-1^3,36^ and, thus, mediates the final passage of leukocytes through the microvascular wall. Of interest, we found that hypoxia and reoxygenation-induced pericytic ICAM-1 expression is also reduced by MA. Similar effects of MA have also been reported for other cell types. Park *et al*.^37^ showed a reduced migration, invasion and adhesion of MA-treated human prostate cancer cells, which was caused by a diminished expression of ICAM-1 and VCAM-1^37^. They further found that this was mediated by a reduced activity of Akt and extracellular signal-related kinases (ERK)^37^. In addition, Li *et al*.^16^ demonstrated that MA inhibits cytokine-induced ICAM-1 expression in pancreatic cancer cells and, thus, alleviates their invasive ability. Taken together, these results indicate that MA acts as a pleiotropic compound targeting the expression of multiple surface adhesion proteins on different cell types.

Hypoxia activates different signaling pathways^38^, including NFκB^5–7^. Furthermore, it has been reported that MA inhibits the NFκB pathway by preventing IκBα phosphorylation, which, in turn, reduces nuclear localization, phosphorylation and DNA-binding of p65^16,25^. Accordingly, we investigated whether the herein observed reduced expression of the adhesion molecules E-selectin, ICAM-1 and VCAM-1 is mediated by the inhibition of NFκB. By means of immunofluorescence microscopy we could demonstrate that MA inhibits the shuttling of the transcription factor p65 into the nucleus of both endothelial cells and pericytes. In addition, it has been shown that MA supresses the activity of PKC^19,39^. This kinase is also crucially involved in hypoxia-induced signaling^40,41^. In addition, PKC is upstream of the NFκB pathway^42^ and, thus, it can be speculated that the downregulatory effect of MA on adhesion molecule expression is caused by a dual inhibition of NFκB signaling and PKC activity.

Our *in vitro* results were confirmed in the dorsal skinfold chamber model, which is suitable for the analysis of leukocyte-endothelial cell interactions under inflammatory conditions^43,44^. Of interest, we detected a higher number of rolling leukocytes in post-ischemic venules of MA-treated mice when compared to controls. This may be explained by our observation that MA reduces the expression of E-selectin on endothelial cells. E-selectin mediates slow leukocyte rolling and contributes in some way to leukocyte adhesion. This reduces leukocytes from the rolling pool. Indeed, Kunkel *et al*.^31^ showed that loss of E-selectin increases the rolling of leukocytes in microvessels. Moreover, we found that venular leukocyte adhesion is significantly diminished after MA treatment. In line with our *in vitro* results, additional Western blot analyses demonstrated that this was due to a reduced endothelial expression of ICAM-1 and VCAM-1.

Studies have reported that MA is capable of reducing pro-inflammatory cytokines, including TNF-α and interleukin (IL)-6^18,19^. These cytokines stimulate the recruitment of different immune cells into ischemic tissue^45,46^. Therefore, we further assessed the number of MPO-positive neutrophilic granulocytes and CD68-positive macrophages infiltrating the tissue of the dorsal skinfold chamber by means of immunohistochemistry. We found that the number of these cells was significantly lower in MA-treated animals when compared to vehicle-treated controls.

Besides its inhibitory action on cytokine release, MA has also been shown to interact with secretory phospholipase A2-IIA (sPLA2-IIA), one of the key enzymes causing lipoprotein modification and vascular inflammation^47^. Of interest, Yap *et al*.^48^ reported that MA suppresses sPLA2-IIA-mediated monocyte migration. Hence, we suggest that this may have additionally contributed to the herein observed inhibition of macrophage recruitment in the dorsal skinfold chamber tissue of MA-treated animals.

In light of the fact that MA exhibits an anti-oxidative activity^17,21,22^, we further analyzed the expression of the NO-producing enzyme eNOS within the tissue of vehicle- and MA-treated dorsal skinfold chambers. We found that high doses of MA significantly increase the expression of eNOS when compared to controls. These results are in line with Li *et al*.^22^ reporting an elevated activity of eNOS in MA-treated endothelial cells. NO is an important signaling molecule, which is involved in the regulation of HO-1 gene expression^49^. Moreover, it is known that MA induces the nuclear accumulation of the transcription factor nuclear factor erythroid 2-related factor 2 (Nrf2), which, in turn, transcriptionally activates the expression of HO-1^21^. Accordingly, we also measured an increased expression of this heat shock protein in MA-treated animals. The anti-oxidative activity of MA was further verified by a significantly reduced number of 8-OHDG-positive cells within the dorsal skinfold chamber tissue of MA-treated animals, indicating that the phytochemical compound prevents oxidative DNA damage.

Finally, although not further investigated in the present study, it may be speculated that the herein observed effects of MA are partially mediated by reversible blockade of the mitochondrial respiratory chain as another potential mechanism of action. In fact, several studies could demonstrate that the suppression of electron transport during ischemia by complex I inhibitors protects mitochondria against ischemic damage and attenuates the mitochondrial release of ROS during reperfusion^50–52^. To test our hypothesis it should first be clarified whether complex I is actually a target of MA by determining the compound’s binding affinity to the hydrophobic site of the enzyme and its inhibitory action on enzyme activity. If this succeeds, it would be interesting to compare in additional studies the protective effectiveness of MA against I/R-induced inflammation and tissue injury with that of conventional complex I inhibitors.

There is considerable interest for the application of MA in clinical practice. MA has already been shown to protect cortical neurons against oxygen and glucose deprivation damage by inhibition of caspase cleavage and ROS formation^53^. More recently, Quian *et al*.^54^ reported that MA extends the therapeutic window of the NMDA receptor antagonist MK-801 in a model of ischemic stroke. The present study now demonstrates that MA inhibits I/R-induced inflammation by downregulation of NFκB-mediated surface adhesion molecule expression. Our results further show that MA treatment prevents oxidative DNA damage. This anti-oxidative activity of MA may have markedly contributed to the suppression of NFκB signaling. Indeed, ROS can activate NFκB through alternative IκBα phosphorylation and influence the DNA binding properties of the NFκB proteins themselves^55^.

Considering the fact that MA exhibits a favorable safety profile in toxicity studies^13,56^, these findings indicate that this phytochemical compound may represent an attractive candidate for the development of novel adjuvant treatment strategies against I/R-induced tissue injury.

## Methods

### Chemical and biological reagents

Endothelial cell basal medium and pericyte growth medium were purchased from PromoCell (Heidelberg, Germany). Dulbecco’s modified Eagle’s medium (DMEM) low glucose was from Lonza (Cologne, Germany). Human TNF-α was from Provitro (Berlin, Germany). MA was purchased from Santa Cruz Inc. (Heidelberg, Germany). Fluorescein isothiocyanate-labeled dextran 150,000, rhodamine 6 G and peroxidase-labeled-streptavidin were purchased from Sigma-Aldrich (Munich, Germany). Mayer’s hemalaun solution was from Merck (Darmstadt, Germany). 3-Amino-9-ethylcarbazole was obtained from Abcam (Cambridge, UK). Ketamine (Ursotamin) was from Pharmacia GmbH (Erlangen, Germany) and xylazine (Rompun) was from Bayer (Leverkusen, Germany).

### Antibodies

The anti-p65 antibody (sc-372), the anti-PDGFR-β antibody (sc-432), the anti-β-actin antibody (sc-517582) and the anti-NG2 antibody (sc-166251) were obtained from Santa Cruz Inc. The antibodies anti-CD54 (ICAM-1) (555511), anti-CD62E (E-selectin) (551145), anti-CD106 (VCAM-1) (555647) and IgG1-κ Isotype Control (555749) were purchased from BD Biosciences (Heidelberg, Germany). Peroxidase-labeled anti-rabbit antibody (NIF 824) and peroxidase-labeled anti-mouse antibody (NIF 825) were obtained from GE Healthcare (Freiburg, Germany). The anti-HO-1 antibody (ADI-SPA-895) was from Enzo Life Sciences (Lörrach, Germany). The anti-α-tubulin antibody (ab56676), the anti-myeloperoxidase (MPO) antibody (ab9535), the anti-ICAM-1 antibody (ab124760), the anti-8-OHDG antibody (ab10802), the anti-eNOS antibody (ab5589), the anti-VCAM-1 antibody (ab134047), the anti-CD68 antibody (ab1252212) and the secondary biotinylated goat anti-rabbit antibody (ab64256) were purchased from Abcam.

### Cell culture

Human placenta-derived pericytes and HDMEC were purchased from PromoCell and cultivated at 37 °C under a humidified 95/5% (vol/vol) mixture of air and CO_2_. Cells were passaged at a split ratio of 1:3 after reaching confluence. All experiments were carried out with confluent cells between the third and seventh passage.

### Hypoxia and reoxygenation

HDMEC and pericytes were treated with different concentrations of MA or vehicle (DMSO) and cultivated in DMEM (low glucose without fetal calf serum) under hypoxic conditions (95% N_2_, 4% CO_2_ and 1% O_2_) for 16 h. Afterwards, the medium was replaced by endothelial cell basal medium or pericyte growth medium and the cells were cultivated for additional 4 h or 24 h under normoxia (95% air and 5% CO_2_) prior to the different analyses.

### WST-1 assay

A WST-1 assay (Roche, Mannheim, Germany) was used to evaluate the mitochondrial activity of HDMEC and pericytes as described previously in detail^44^.

### LDH assay

A LDH assay (Cytotoxicity Detection KitPLUS, Roche) was used to analyze the viability of HDMEC and pericytes as previously described in detail^44^.

### Annexin V/PI assay

Apoptotic and necrotic cells were detected by an Annexin V/PI detection kit (Invitrogen, Karlsruhe, Germany). Briefly, after hypoxia and reoxygenation cells were incubated at room temperature with Annexin V and PI for 30 min. The mean fluorescence intensity (MFI) of 5,000 cells was analyzed in the FL-1 and FL-2 channel by a FACScan flow cytometer (Becton Dickinson, San Jose, CA, USA) using the CellQuest software.

### Western blot analysis

Cytoplasmic and nuclear extracts from HDMEC and pericytes as well as dorsal skinfold chamber tissue extracts were generated as described previously in detail^57^. Proteins of cell extracts were separated through a 12.5% SDS polyacrylamide gel and transferred onto a polyvinylidene difluoride (PVDF) membrane. The membrane was incubated in 5% dry milk in phosphate-buffered saline (PBS) (0.1% Tween20) for 1 h and exposed to anti-p65, anti-ICAM-1, anti-VCAM-1, anti-eNOS, anti-HO-1, anti-β-actin, anti-α-tubulin and anti-nucleolin antibodies, which were diluted (1:500) in PBS (0.1% Tween20) containing 1% dry milk. After incubation of the membrane with a peroxidase-coupled secondary antibody (anti-rabbit 1:30,000 or anti-mouse 1:10,000) for 1 h, protein expression was detected by means of luminol-enhanced chemiluminescence (ECL; GE Healthcare).

### Flow cytometry

HDMEC and pericytes were cultivated under hypoxia and reoxygenation in medium supplemented with MA or vehicle (DMSO). Subsequently, the cells were washed in PBS and harvested by incubation with trypsin/EDTA (0.25% (w/v) trypsin, 0.02% EDTA) for 5 min at 37 °C. Subsequently, the cells were incubated with the phycoerythrin (PE)-labeled primary antibodies E-Selectin, ICAM-1, VCAM-1 and the PE-labeled IgG control antibodies for 30 min at room temperature. Then, HDMEC and pericytes were washed in PBS and the MFI of 5,000 cells was analyzed in the FL-2 channel by a FACScan flow cytometer (Becton Dickinson) using the CellQuest software.

### Immunofluorescence microscopy

HDMEC and pericytes were seeded on coverslips and treated with different concentrations of MA or vehicle (DMSO) and exposed to hypoxia and reoxygenation. Then, the cells were fixed in PBS (3.7% formalin) for 10 min and subsequently permeabilized in PBS (0.2% Triton X-100) for 30 min. Afterwards, the cells were blocked in PBS (2% BSA) for further 30 min at room temperature. HDMEC and pericytes were incubated with anti-p65 antibodies (1:50) and the secondary anti-rabbit antibodies (1:250). Subsequently, the cells were sealed with mounting media and analyzed by fluorescence microscopy (BX60; Olympus, Hamburg, Germany).

### Animals

In this study, male and female BALB/c mice with a body weight of 20–30 g were used. They were bred and housed in open cages in the conventional animal facility of the Institute for Clinical & Experimental Surgery (Saarland University, Germany). Housing was performed in a temperature-controlled environment under a 12 h/12 h light-dark cycle. The animals had free access to standard pellet chow (Altromin, Lage, Germany) and water. The experiments were approved by the local governmental animal protection committee (Landesamt für Verbraucherschutz, Abteilung C Lebensmittel- und Veterinärwesen, Saarbrücken, Germany; permit number: 15/2014) and were conducted in accordance with the European legislation on protection of animals (Guide line 2016/63/EU) and the National Institutes of Health Guidelines for the Care and Use of Laboratory Animals (http://oacu.od.nih.gov/regs/index.htm. Eighth Edition; 2011).

### Dorsal skinfold chamber model

The dorsal skinfold chamber served as model to analyze the effects of MA on I/R-induced inflammation. For the implantation of the chamber, the mice were anesthetized by intraperitoneal (i.p.) injection of ketamine (75 mg/kg) and xylazine (15 mg/kg). The chamber was prepared as described previously in detail^58^. In brief, two symmetrical titanium frames were implanted on the extended dorsal skinfold of the animals. One layer of the skin was removed in a circular area of 15 mm in diameter and the remaining layers consisting of the cutis, subcutaneous tissue and striated skin muscle were covered with a glass coverslip. After surgery, mice were allowed to recover for 72 h.

Ischemia was induced as previously described in detail^59^. Briefly, the striated muscle tissue of the chamber was gently pressed for 3 h against the cover slip of the observation window using an adjustable screw. The pressure of 40 mmHg was sufficient to occlude all arteriolar, capillary and venular blood vessels within the chamber, thus, garnished complete ischemia. Subsequently, the screw was removed to restore blood perfusion. To investigate the effect of MA on leukocytic inflammation, 8 animals were treated with 10 mg/kg MA or 20 mg/kg MA i.p. (dissolved in 50 µL DMSO) 19 h and 1 h before the induction of ischemia. As controls we used 8 vehicle-treated mice. For Western blot analyses of the dorsal skinfold chamber tissue after 3 h ischemia and 3 h reperfusion, additional animals were treated with 20 mg/kg MA i.p. (n = 3) or vehicle (n = 3) 19 h and 1 h before the induction of ischemia.

### Intravital fluorescence microscopy

For intravital fluorescence microscopy the animals received a retrobulbary i.v. injection of 0.05 mL 5% FITC-labeled dextran 150,000 to enhance the contrast of blood-perfused microvessels. Moreover, they received 0.05 mL 0.1% rhodamine 6 G i.v. for *in situ* staining of leukocytes. Subsequently, the mice were fixed on a plexiglas stage and the dorsal skinfold chamber was horizontally positioned under a Zeiss microscope (Zeiss, Oberkochen, Germany) equipped with a 100 W mercury lamp attached to a blue (excitation wavelength: 450–490 nm/emission wavelength: >515 nm) and green (530–560 nm/>585 nm) filter block. The microscopic images were recorded by a charge-coupled device video camera (FK6990; Pieper, Schwerte, Germany) connected to a monitor (Trinitron; Sony, Tokyo, Japan) and DVD system (DVD-HR775; Samsung, Eschborn, Germany)^60^.

The microscopic images were analyzed by means of the off-line analysis system CapImage (Zeintl, Heidelberg, Germany). Leukocyte-endothelial cell interactions, microhemodynamic parameters, functional capillary density and macromolecular leakage were assessed in 8 randomly selected venules per chamber. Measurements were performed under baseline conditions before injection of MA or vehicle 19 h prior to ischemia as well as 0.5 h, 3 h and 24 h during post-ischemic reperfusion of the tissue^43,59^. Finally, the dorsal skinfold chamber tissue was used for additional immunohistochemical analyses.

### Immunohistochemistry

Formalin-fixed specimens of the chamber tissue were embedded in paraffin for the cutting of 3 µm-thick sections. The sections were stained with a polyclonal rabbit anti-mouse antibody against the neutrophilic granulocyte marker MPO or against the macrophage marker CD68 followed by a biotinylated secondary goat anti-rabbit IgG antibody. The biotinylated antibody was detected by peroxidase-labeled-streptavidin. 3-Amino-9-ethylcarbazole was used as chromogen. The sections were counterstained with Mayer’s hemalaun. As negative controls we used sections solely incubated with the secondary antibody. MPO-positive and CD68-positive cells were counted in 10 high power fields (HPF) per section and are given as cells/HPF. For the detection of 8-OHDG, the sections were stained with a polyclonal goat anti-mouse antibody against anti-8-OHDG followed by a secondary coupled fluorescence antibody. As negative controls we used sections solely incubated with the secondary antibody. 8-OHDG-positive cells were counted in 10 HPF per section and are given as cells/HPF.

### Statistical analysis

After testing the data for normal distribution and equal variance, differences between two groups were analyzed by the unpaired Student’s t-test and differences between multiple groups were analyzed by one-way ANOVA followed by the appropriate post hoc test, including the correction of the alpha error according to Bonferroni’s probabilities (SigmaPlot 13.0; Jandel Corporation, San Rafael, CA, USA). To test for time effects in individual groups, ANOVA for repeated measures was applied followed by Bonferroni-t-test. All values are expressed as mean ± standard deviation (SD). Statistical significance was accepted for P < 0.05.

## Supplementary information

Supplementary Info File #1

## Data Availability

All data generated or analyzed during this study are included in this published article
