# Supplementary material for: Maslinic acid alleviates ischemia/reperfusion-induced inflammation by downregulation of NFκB-mediated adhesion molecule expression
Source: Sci Rep. 2019 Apr 16;9:6119. doi: 10.1038/s41598-019-42465-7 (PMC6467883; doi:10.1038/s41598-019-42465-7)
Supplement: Supplementary file 1 — Supplementary Info File #1 [file 41598_2019_42465_MOESM1_ESM.pdf]

Maslinic acid alleviates ischemia/reperfusion-induced inflammation by downregulation of NFκB-mediated adhesion molecule expression

Emmanuel Ampofo[1,#,*], Julian J. Berg[1,#], Michael D. Menger[1] and Matthias W. Laschke[1]

[1]*Institute for Clinical & Experimental Surgery, Saarland University, 66421 Homburg/Saar, Germany*

[#]Contributed equally to this work

***Address for correspondence:**
Emmanuel Ampofo, Ph.D.
Institute for Clinical & Experimental Surgery
Saarland University
66421 Homburg/Saar
Germany

phone: +49 6841 162 6561
fax: +49 6841 162 6553
E-mail: emmanuel.ampofo@uks.eu

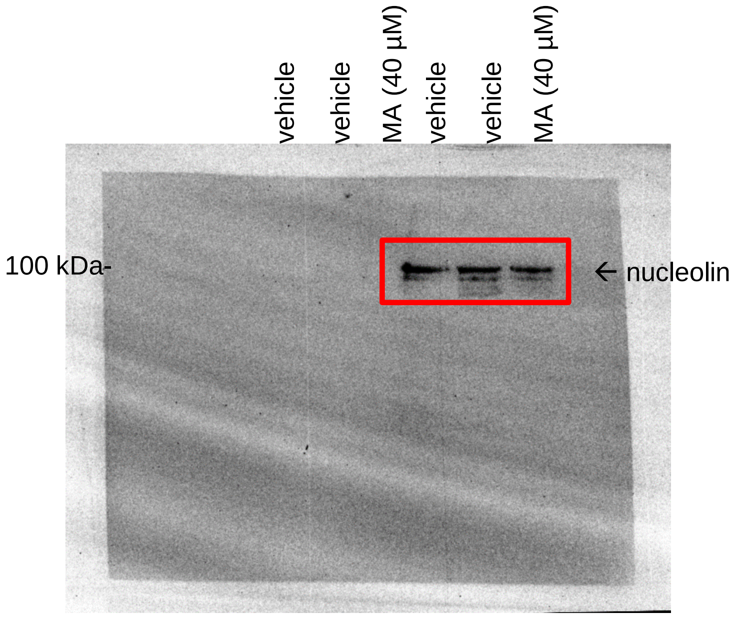

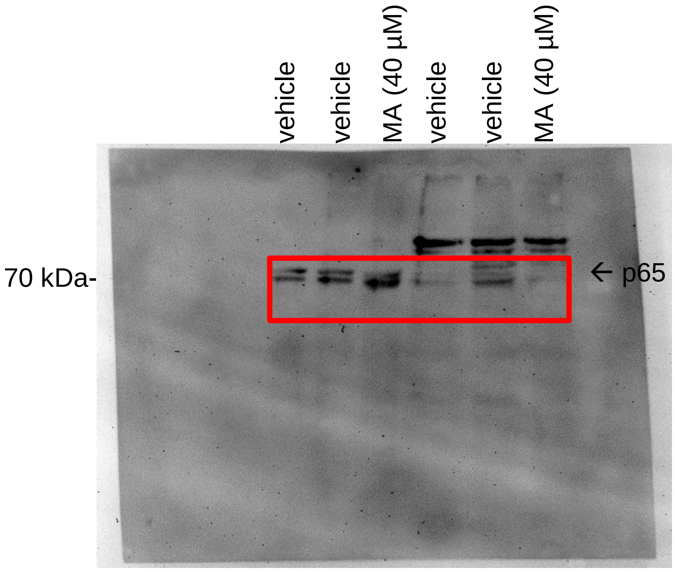

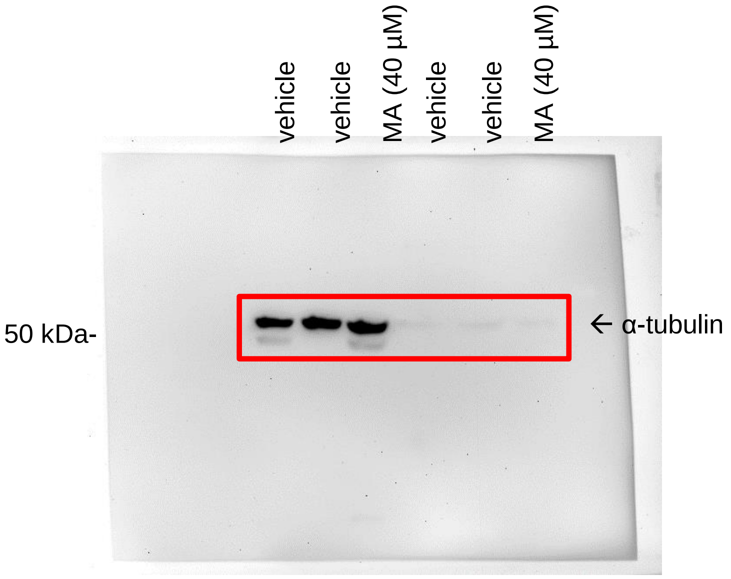

Uncropped images of blots presented in figure 2

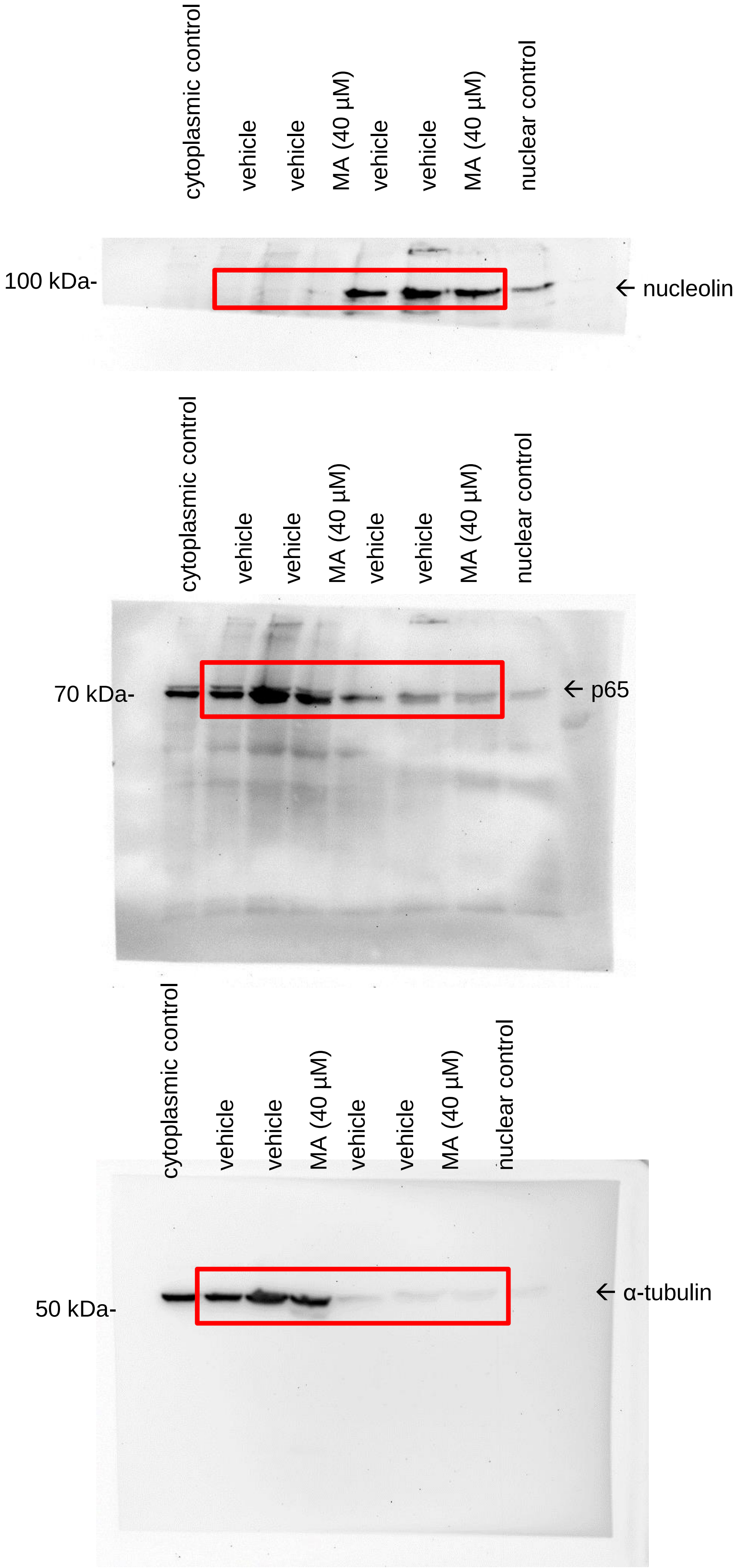

Uncropped images of blots presented in figure 3

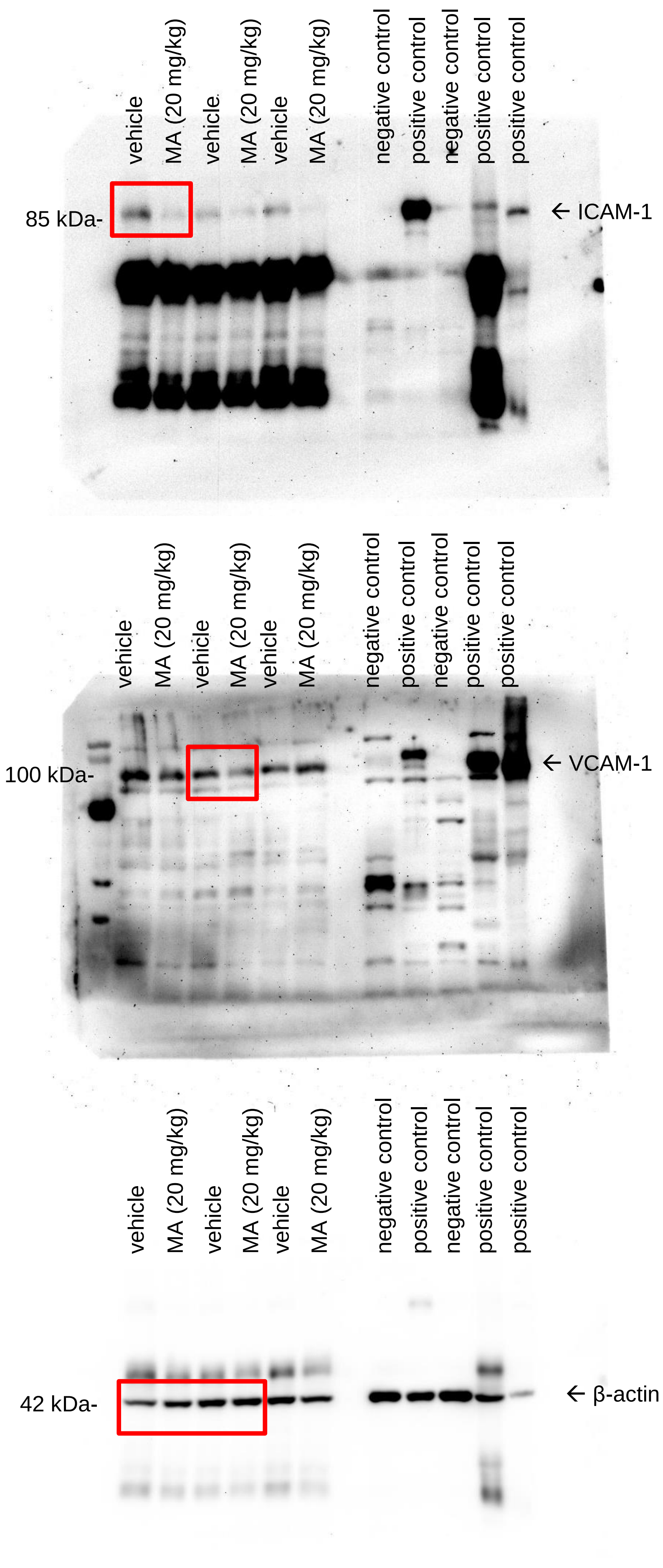

Uncropped images of blots presented in figure 5

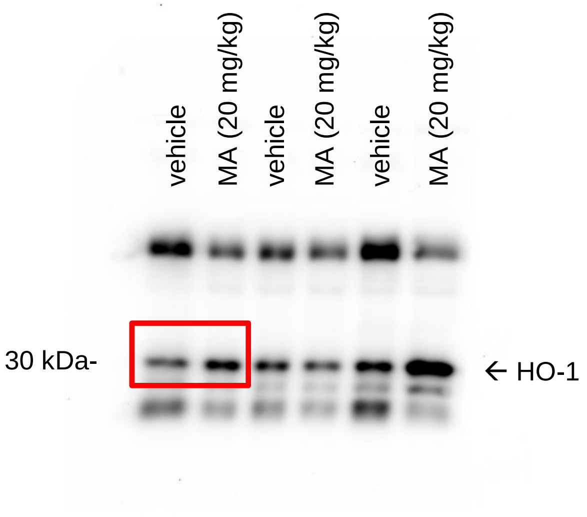

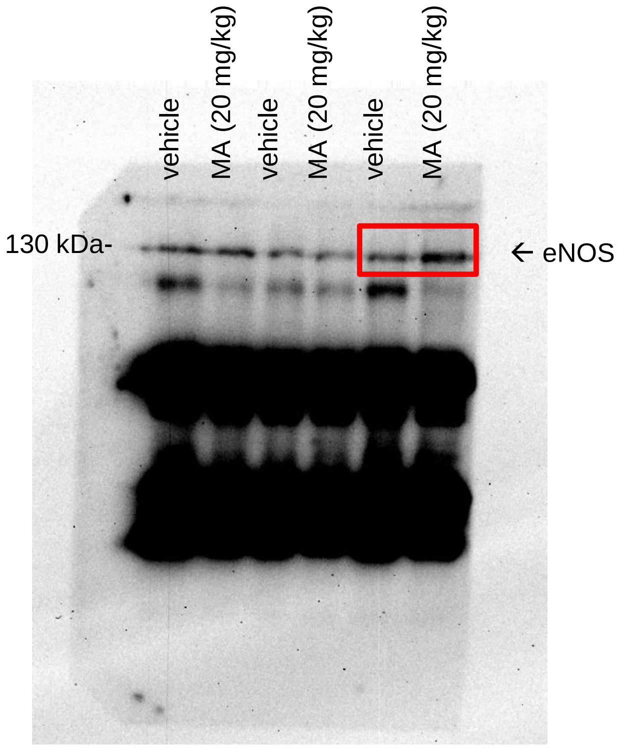

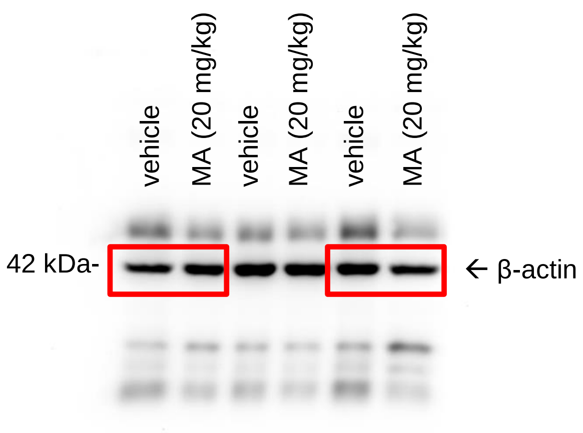

Uncropped images of blots presented in figure 6
